# Remobilized volcanogenic sediments boost photophysiological activity in three symbiotic reef-building coral species

**DOI:** 10.1098/rsos.252217

**Published:** 2026-10-07

**Authors:** Frank Förster, Christine Ferrier-Pagès, Cécile Rottier, Allan Fries, Eric Achterberg, Tom Sheldrake

**Affiliations:** ^1^ Department of Earth Sciences, University of Geneva, Geneva, Switzerland; ^2^ Equipe Biologie Corallienne, Centre Scientifique de Monaco, Monaco-Ville, Monaco; ^3^ GEOMAR Helmholtz Centre for Ocean Research Kiel, Kiel, Schleswig-Holstein, Germany

**Keywords:** coral physiology, volcanology, trace metals, experimental design, coral reefs

## Abstract

Coral reefs that surround volcanic islands are continually influenced by run-off carrying remobilized volcanogenic sediments. Although unaltered volcanic ash is known to supply trace metals that enhance coral holobiont productivity, the impacts of long-term exposure to weathered tephra, a far more likely scenario in island settings, are less understood. To assess possible effects of tephra exposure on coral physiology, we conducted controlled culture experiments with three scleractinian corals (*Stylophora pistillata*, *Turbinaria reniformis* and *Pocillopora damicornis*) exposed to weekly additions of 50 g of untreated tephra, collected from the volcanic island of St. Vincent, and its leachate over a six-week period. All exposed colonies experienced optimized photosynthetic light-harvesting processes, while most other physiological parameters were not affected. Remobilized tephra supplies hormetic concentrations of water soluble trace metals, with high-resolution inductively coupled plasma mass spectrometer (HR-ICP-MS) analyses showing significant increases in seawater trace metal (V, Mn, Fe, Co, Ni, Cu, Cd and Pb) concentrations. This highlights tephra's previously underrecognized contribution to the island mass effect affecting corals.

## 1. Introduction

Coral reefs are prominent marine ecosystems that surround tropical volcanic islands [1,2]. However, the impacts of volcanic eruptions and volcanoclastic sediments on these ecosystems, and particularly on scleractinian corals, the reef builders, are understudied. This gap may stem from the unpredictable and infrequent nature of volcanic eruptions [3], as well as hazardous or remote sampling conditions [4,5], which limit empirical observations.

Most observations have focused on large-scale volcanic eruption events and their associated primary hazards (such as tephra fallout [4,6,7], pyroclastic density currents (PDCs) or lava flows [8]), which can cause severe environmental damage (e.g. [9,10]). Deposited tephra (i.e. airborne rock fragments produced during an eruption) can completely bury coral reefs and preserve entire reef communities in the geological record [11]. Long-term environmental changes are sustained following eruptions, prolonging impacts on terrestrial and marine ecosystems beyond the active volcanism period (e.g. [5,8,10]). Initial impacts are frequently extended in space and time as remobilization by water and wind causes secondary transport to areas wider than those originally affected [12]. However, the long-term effects of volcanic eruptions on corals remain understudied [5,13]. For instance, the enhanced coastal sediment influx, owing to ongoing terrestrial erosion of unconsolidated volcanic material, may occur over thousands of years following specific eruption events [14]. Coastal influx of volcanogenic sediments is sourced from pyroclastic deposits surrounding a volcano, which are commonly remobilized during intense precipitation events, such as tropical storms [15,16].

Further investigation into the impacts of remobilized volcanogenic sediments is warranted, given that recent laboratory studies on the scleractinian coral *Stylophora pistillata* reported that pristine volcanic ash can leach physiologically important micronutrients (i.e. manganese and iron) at biologically relevant concentrations [17,18]. These micronutrients not only affect the coral metal household [17] but are also essential for the functioning of the photosynthetic processes of the Symbiodiniaceae [19], which are dinoflagellates living in symbiosis with corals and which largely cover the energetic needs of the coral host [20]. Therefore, positive effects of ash on the symbiont's photosynthetic apparatus have been reported, with increased gross and net photosynthesis rates, as well as significant increases in the effective and maximum quantum yield of photosystem II [18]. Altogether, these observations suggest that the Symbiodiniaceae are limited in essential micronutrients for an optimal photosynthetic functioning.

To date, studies have only focused on one scleractinian coral species (*S. pistillata*) and have only tested its response to pristine volcanic ash. Pristine volcanic ash represents fine, fragmented volcanic material erupted into the atmosphere and collected immediately upon deposition on land. These deposits are uniquely fertile owing to the soluble metal salt encrustations on ash particles that rapidly dissolve upon contact with water (e.g. [21–23]). Consequently, the supply of micronutrients from wind- and water-driven remobilization of weathered tephra deposits is probably lower per unit mass than from pristine ash. However, the volume of terrestrial pyroclastic material available for remobilization can be much larger than that of ash fallout deposits. For example, this notably includes voluminous PDC deposits, which were several metres thick following the 2021 eruption of La Soufrière on St. Vincent [24]. In coastal regions, remobilized material, therefore, contributes more to the total tephra input than freshly erupted ash. Such tephra exposure can alter the ambient chemical composition of seawater, yet the extent of these changes and whether trace elements are leached in bio-accessible forms for marine biota remain unclear.

To assess the physiological impacts of prolonged exposure of corals to moderate amounts of weathered tephra, we conducted controlled six-week coral culture experiments on three species of symbiotic scleractinian corals (*S. pistillata*, *Pocillopora damicornis* and *Turbinaria reniformis*). The coral nubbins were exposed to identical concentrations of particulate tephra either directly (i.e. tephra particles (treatment II)) or indirectly (i.e. via a filtered tephra–seawater solution (treatment III)) and compared to control nubbins (treatment I). Such research is critical to understand biosphere–geochemosphere interaction, especially since coral reefs become increasingly fragile under anthropogenic pressures such as global warming and ocean acidification [25].

## 2. Material and methods

### 2.1. Tephra samples

In March 2024, pyroclastic deposits (2.32 kg of tephra) were sampled at the crater rim of the La Soufrière volcano (13°19.6416 N, 061°10.8133 W; 984 m above sea level) on the volcanic island of St. Vincent (Eastern Caribbean). These deposits were formed during the explosive phase of the eruption of the La Soufrière volcano between 9 and 22 April 2021, in which a bulk volume of approximately 120 × 10^6^ m^3^ of tephra was emitted [26]. The tephra had been exposed to weathering and rainfall during the three years between deposition and sampling. Powder X-ray diffraction analysis revealed calcium-rich plagioclase as the dominant mineral phase (greater than 80%), followed by pyroxene (approx. 9%), cristobalite (approx. 5%) and iron oxides (less than 5%) [27]. The silica (SiO_2_) content of approximately 54–56 wt.% and a total alkali content (Na_2_O + K_2_O) of approximately 3–4 wt.% classify the 2021 tephra products as basaltic andesite [28], with major and minor elements broadly identical between deposited tephra types [27]. The same study also reported a comparable glass content of approximately 30% in the tephra from St. Vincent. The oven-dried (for 1 week at 40°C) but otherwise untreated tephra (unsieved and uncrushed) was stored in plastic zipper bags for transportation, and it was used without further sample preparation in the coral exposure experiments.

#### 2.1.1. Particle size distribution

The particle size distribution of the tephra sample was determined by combining dry sieving analysis for particles greater than 500 µm and laser diffraction performed with the Bettersizer S3 Plus for particles with a diameter less than 500 µm. The complete particle size distribution was obtained from merging the laser diffraction results with those obtained from sieving. For that purpose, the particle density was assumed to be constant regardless of the particle size, thus allowing us to establish an equivalence between volume percentages (as acquired from laser diffraction for the fine particles) and weight percentages (as obtained from sieving) (supplementary material, table S1). The Bettersizer measurements showed a variability of less than 5% (*n* = 3). Characteristic particle diameters, including the median (D_50_) and the 84th and 16th percentiles (D_84_ and D_16_) of the cumulative particle size distribution, were calculated by logarithmic interpolation between the two sieve sizes (*d*_1_ and *d*_2_) that bracket the target cumulative percentage, following Bunte & Abt [29]:

$$\begin{array}{r} \log(D_{50})=\log(d_{1})+\frac{50-P_{1}}{P_{2}-P_{1}}\left(\log(d_{2})-\log(d_{1})\right) \end{array}$$

with the sieve sizes (*d*_1_ and *d*_2_) immediately above and below the 50% passing and *P*_1_ and *P*_2_ the corresponding cumulative percent passing values (supplementary material, table S1). The sorting coefficient (*σ*), representing the spread of the particle size distribution, was calculated according to Inman [30]:

$$\begin{array}{r} \sigma =\frac{D_{84}-D_{16}}{2} \end{array}$$

### 2.2. Experimental set-up

Coral culture experiments were performed in a controlled aquarium set-up using lab-grown microcolonies (from now on ‘nubbins’) of three scleractinian corals (*S. pistillata*, *T. reniformis* and *P. damicornis*). Coral species were selected for their ecological resilience and functional importance. While *S. pistillata* and *P. damicornis* are considered fast-growing, opportunistic, early-successional species capable of colonizing primary substrata [31–33], *T. reniformis* grows slower but is relatively stress-tolerant owing to its higher biomass and total energy reserves [34]. A total of 48 nubbins of *S. pistillata*, 36 nubbins of *T. reniformis* and nine nubbins of *P. damicornis*, each one a few cm in size, were cut from their mother colonies with a bone cutter. While nubbins of the branching corals *S. pistillata* and *P. damicornis* were hung on nylon fish threads, *T. reniformis* nubbins were placed at the bottom of the aquaria. Nubbins were allowed to heal from cutting during four weeks and were maintained in several aquaria under the following culture conditions: irradiance of 200 ± 15 µmol photons m^−2^ s^−1^ with a photoperiod of 12 h d^−1^, 26 ± 0.39°C water temperature, a pH_SWS_ of 8.19 ± 0.11, constant seawater renewal (i.e. open system) and feeding with *Artemia salina* nauplii twice a week. Coral nubbins were then evenly distributed over six 30 l experimental tanks, where they were maintained over the six-week experimental period. Each tank contained eight nubbins of *S. pistillata*, six nubbins of *T. reniformis* and one or two nubbins of *P. damicornis* per tank. The experimental set-up consisted of eight 30 l tanks in total, with two coral-containing tanks assigned to each of the three treatments (figure 1). Treatment I served as control in which corals received no tephra. In treatment II, 50 g of unsieved tephra was added every week (on Mondays) directly to the coral tanks. In treatment III, corals were indirectly exposed to tephra. For this, 50 g of unsieved tephra was added once per week to two separate tanks that did not contain corals. These tanks acted as tephra leachate reservoirs. Water from these reservoirs was supplied to the coral-containing tanks using a system of peristaltic pumps connected to transfer tubes and a 10 µm meshed nylon net (figure 1). The mesh prevented all tephra (larger than 10 µm) from entering the coral tanks of treatment III. The flow rate from the tephra reservoir to the coral tanks in treatment III was maintained for 9 h a day (Mondays to Fridays). Overnight and on the weekends, the seawater inlet flow was restored to the coral tanks, and the pump was turned off. To ensure comparability across treatments, the seawater inlet flow rates for the control (treatment I) and the particulate tephra treatment (treatment II) were adjusted to match the peristaltic pump rate. Across all treatments, the average seawater inlet flow was measured at 138 ± 14 ml min^−1^. Flow rates were recorded each morning throughout the six-week-long experiment.

**Figure 1.**
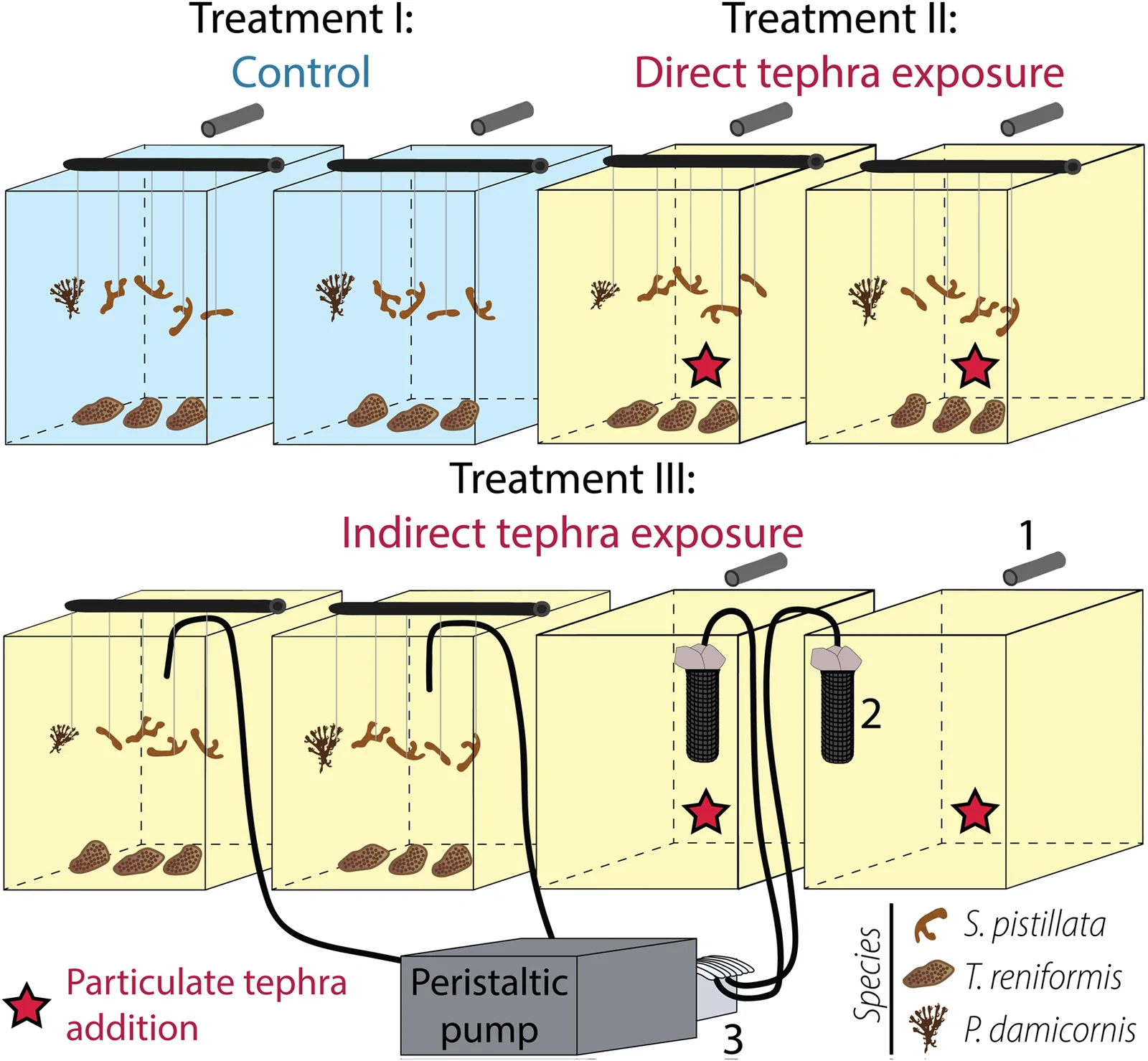
Experimental set-up of the six-week tephra exposure study with tanks under three treatments (I – control, II – direct tephra exposure and III – indirect tephra exposure). Each treatment included two coral tanks, with particulate tephra addition (50 g week^−1^) symbolized by a red star. Each coral tank contained two rows of corals, totalling eight nubbins of *Stylophora pistillata*, six nubbins of *Turbinaria reniformis* and one or two *Pocillopora damicornis* per tank (one nubbin in the left-hand tank and two in the right-hand tank of each treatment). Only the front row of corals is illustrated. Highlighted features: (1) seawater inlet supplying continuously 5 µm filtered seawater pumped from 40 m depths to the experimental tanks, (2) 10 µm nylon mesh surrounding a plastic housing with cartridge tubes to prevent particulate tephra from entering coral tanks in treatment III, and (3) Masterflex® L/S 7550-22 peristaltic pump with 7519-06 cartridge pump head delivering tephra leachate to coral tanks of treatment III at a flow rate of approximately 140 ± 10 ml min^−1^.

Coral nubbins across all treatments were reared at the above culture conditions, except that feeding with *Artemia* nauplii was stopped one week before the experiments started to prevent interaction between tephra-derived metals and the nauplii-derived metals. The water was maintained at a constant temperature using an automated temperature controller (Enoleo Monaco, Monaco), and water circulation in the tank was ensured using submersible pumps (Micro-Jet, MC320, 320 l h^−1^; Newa®). All tanks were cleaned weekly on Fridays to remove the tephra and algae.

Given the bottom tank area of 0.088 m^2^, the addition of 50 g tephra corresponds to a weekly deposition of approximately 0.57 kg m^−2^, equivalent to 11.4 mg cm^−2^ d^−1^. This exposure scenario falls within the range reported in other sediment exposure studies (reviewed in Tuttle & Donahue [35]). Moreover, a loading of approximately 0.57 kg m^−2^ represents a realistic scenario for coral reefs in the Coral Triangle, where nearly 40% of reefs are projected to experience tephra deposition of 1 kg m^−2^ at recurrence intervals of several hundred years, with more frequent exposure to lower loads (based on ash fall exposure projections by Fischer [36]). Although the selected tephra quantity and frequency were not based on a precise biogeochemical justification, given that deposition and erosion rates vary greatly (i.e. with eruption type, duration, surface topography and distance from a volcano), the chosen dose represents a plausible lower-range exposure scenario.

Once a week (on Mondays), at the end of the day, after 9 h of exposure, 10–15 ml of seawater was sampled from each coral-containing aquarium (*n* = 6) using a 50 ml polypropylene (PP) syringe, equipped with a 0.22 µm PES syringe filter (Fisherbrand®). Both were rinsed three times with 0.45-µm filtered seawater before usage. The collected solution was transferred to metal-free PP tubes, acidified to 0.5 M HNO_3_ with ultrapure nitric acid (NORMATOM®) and stored at 4°C.

### 2.3. High-resolution inductively coupled plasma mass spectrometer analysis of seawater metal concentration

Seawater samples collected on the second, third and sixth Monday (totalling *n* = 18) after the experiment began were analysed using a high-resolution inductively coupled plasma mass spectrometer (HR-ICP-MS) (Element XR^TM^, Thermo Scientific) at GEOMAR, Helmholtz Centre for Ocean Research Kiel. Samples were either diluted 10× or 30× before preconcentration of trace metals and removal of the seawater matrix by seaFAST [37]. Analytical accuracy was assessed by routine analysis of a certified reference seawater (NASS-7; *n* = 10, supplementary material, table S2), and trace metal concentrations were quantified using a nine-step calibration curve. Procedural blanks were less than 1 nmol l^−1^ except for Zn (1.44 nmol l^−1^), and method detection limits are reported in table S2 in the supplementary material.

Analysis of NASS-7 [38] showed good accuracy and reproducibility for V, Mn, Fe and Cu (relative bias less than 15%, two-times relative standard deviation (2RSD) less than 35%; supplementary material, table S2); Co exhibited excellent reproducibility (2RSD ≈ 10%), but a moderate bias (approx. 50%). Both Ni and Zn displayed intermediate reproducibility (2RSD = 40–50%) with similar moderate bias (40–60%); Cd and Pb exhibited poor reproducibility (2RSD ≈ 50%) and severe bias (greater than 300%). Thus, these values are reported for transparency but not used for accuracy validation. Trace metal concentrations are presented as nmol l^−1^.

### 2.4. Monitored parameters

#### 2.4.1. Effective quantum yield

On Mondays and Thursdays (twice a week), the effective quantum yield of photosystem II (Φ_PSII_) was measured on *S. pistillata* nubbins (*n* = 4 per tank), *T. reniformis* nubbins (*n* = 3 per tank) and on all nubbins of *P. damicornis* (*n* = 3 per treatment), using a pulse-amplitude-modulated (PAM) fluorometer (DIVING-PAM, Walz®, Germany). The value of Φ_PSII_ was measured at 200 µmol photons m^−2^ s^−1^ on light-adapted nubbins and is calculated as the ratio of chlorophyll-fluorescence parameters *F*′_*m*_ and  *F*_0_ (the maximal and minimal fluorescent yields at ambient light, respectively) according to Genty *et al*. [39]:

$$\begin{array}{r} \Phi_{PSII}=\frac{F_{m}^{\prime }-F_{0}}{F_{m}^{\prime }} \end{array}$$

#### 2.4.2. Skeletal growth rate

Every week, on Wednesdays, the wet mass of all nubbins (42 *S. pistillata* (*n* = 16 per treatment), 36 *T. reniformis* (*n* = 12 per treatment) and nine *P. damicornis* (*n* = 3 per treatment)) was measured using the buoyant weighing technique [40] with a Mettler Toledo XP205 balance (accuracy ± 0.01 mg). The skeletal growth rate was measured as weekly changes in mass, and a percentage increase was calculated relative to the starting mass measured 3 days prior to the experiment. Growth rate is expressed as total percentage increase from the initial nubbin weight and as daily nubbin growth rate (% d^−1^) calculated following Reynaud & Ferrier-Pagès [41].

### 2.5. Photosynthetic parameters

All of the photosynthetic parameters were measured as described by Förster *et al*. [18], at the end of the six-week experiment. A PAM fluorometer (DIVING-PAM, Walz®, Germany) was used to assess the total quantum yield of photosystem II (*F*_v_/*F*_m_), the relative electron transport rate (ETR) and non-photochemical quenching (NPQ), all derived from rapid light curves (RLC), performed on dark-adapted *S. pistillata* (*n* = 8 per treatment), *T. reniformis* (*n* = 6 per treatment) and *P. damicornis* (*n* = 3 per treatment). Gross and net photosynthesis (*P*_gross_ and *P*_net_, respectively) and respiration rates (*R*) were measured on *S. pistillata* (*n* = 8 per treatment) and *T. reniformis* (*n* = 6 per treatment) at 200 µmol photons m^−2^ s^−1^ irradiance (*P*_net_) and in the dark (*R*) at the end of the experiments, using oximetry as described in Förster *et al*. [18]. Values of *P*_gross_, *P*_net_ and *R* were normalized to both the coral surface area and total symbiont count. Surface area for *S. pistillata* and *T. reniformis* nubbins was determined using the single wax dipping technique [42]. Owing to the low number of *P. damicornis* specimens (*n* = 1 or 2 per tank) and the absence of a validated surface area calibration for the wax-weighing method in this species, *P*_net_, *P*_gross_ and *R* were not analysed.

### 2.6. Symbiont density, protein content and chlorophyll content

After the experiments, all coral nubbins were stored at –20°C. Physiological measurements were performed on *S. pistillata* (*n* = 8 per treatment) and *T. reniformis* (*n* = 6 per treatment), but not on *P. damicornis* owing to the low number of specimens per tank (*n* = 1 or 2). Before analysis, coral tissue and symbionts were removed from the skeleton using an airbrush with pressurized air and 0.45 µm filtered seawater. The seawater extract containing both host tissue and symbionts was sequentially homogenized for 20 s using an Ultra-Turrax T25 (Janke & Kunkel (IKA)®), before being aliquoted into three subsamples (500 µl, 5 ml and the remaining volume).

Protein content in the 500 µl subsample was determined as described by Förster *et al*. [18] using the Bradford assay [43], with a calibration curve created from serial dilutions of 2 mg ml^−1^ bovine serum albumin. Protein content (mg) was normalized to surface area (cm^−2^). Centrifugation (10 min at 5000 g and 4°C) of the 5 ml subsamples separated dinoflagellates from host tissues. The symbionts, retained in the pellet after the supernatant was discarded, were resuspended in 5 ml of pure acetone. This mixture was kept in the dark at 4°C for 24 h, after which it was centrifuged again at 5000 g for 15 min at 4°C. Aliquots of 300 µl were transferred in triplicate to a quartz microplate, and absorbance was measured at 630 and 663 nm using a Xenius® SAFAS spectrophotometer. Chlorophyll *a* and *c_2_* concentrations were calculated according to Jeffrey & Humphrey [44] and Ritchie [45] and were normalized to the skeletal surface area and total symbiont amount*.* Symbiont density (number of cells cm^−2^) was determined on three 10 µl aliquots per nubbin from the remaining seawater extract using a LUNA FX7 automated cell counter (Logos Biosystems, South Korea).

### 2.7. Statistical evaluation

Statistical analysis was performed using RStudio [46] with R v.4.2.0 [47]. Differences between experimental treatments were tested for significance using either the non-parametric Kruskal–Wallis test (KW) or one-way analysis of variance (ANOVA) for parametric data. The choice of statistical test was based on assessments of normality and homoscedasticity. Normality was evaluated using the Shapiro–Wilk test, and homoscedasticity was assessed with Levene's test, with the results listed in tables S3 (seawater metal concentration), S6 (chlorophyll-fluorescence parameters) and S8 (physiological data) in the supplementary material. If the KW test indicated a statistically significant result (*p* < 0.05), then Dunn's post hoc test with a Holm–Bonferroni correction [48] was applied to identify significant differences between the treatments. For ANOVA, Tukey's post hoc test was used. Significance levels are represented in the figures as follows: *p*-value > 0.05 (not significant), *p*-value ≤ 0.05 (*), *p*-value ≤ 0.01 (**) and *p*-value ≤ 0.001 (***). For chlorophyll-fluorescence-derived parameters, such as Φ_PSII,_ *F*_v_/*F*_m_, rETR and NPQ, statistical tests were performed on the highest measured values per treatment (Φ_PSII max_) and at the end of the experiment (Φ_PSII end_) or on a single physiologically important irradiance level (i.e. *F*_v_/*F*_m_ at 0 µmol photons m^−2^ s^−1^; rETR_max_ and NPQ_max_ at 1956 µmol photons m^−2^ s^−1^).

## 3. Results

The original data that support the findings of this study are available in the Förster data repository [49]. The temperature and pH of each experimental tank were continuously monitored, with no clear difference observed between tephra-exposed and non-exposed tanks (supplementary material, table S5). Variations in pH remained within the margin of error (supplementary material, fig. S1) and were attributed to instrumental precision limits and natural daily fluctuations. Changes in light irradiance were not quantified. However, visual observations indicated that tephra settled within the first hour in the directly exposed tanks, suggesting negligible effects on light availability (supplementary material, fig. S2). Indirect tephra exposure did not alter light conditions.

### 3.1. Particle size distribution

The particle size distribution, with corresponding cumulative weight percentages of individual fractions, is presented in fig. S3 in the supplementary material. Sieve analysis for particles larger than 500 µm combined with laser diffraction for particles less than 500 µm revealed a median particle size (*D*_50_) of 0.98 mm, calculated by logarithmic interpolation between the 1.0 and 0.71 mm sieves (supplementary material, table S1). The sample sorting coefficient (*σ*) is 1.70 mm. The particle size classifies the tephra as coarse ash to fine lapilli, with less than 5% of the sample categorized as very fine ash (less than 63 µm in diameter [50,51]) and less than 1% of the mass composed of particles smaller than 10 µm (i.e. the filter size used in treatment III).

### 3.2. Seawater metal concentration

Both direct and indirect tephra exposure (treatments II and III) led to significant increases in the seawater trace metal concentrations except for Zn (table 1 and supplementary material, table S4). These concentrations exceeded background seawater concentrations in the tanks by up to 10 times (i.e. for Ni in direct tephra-exposed seawater). The largest increases in treatment II compared to treatment I were observed with Ni (10.6-fold), Co (9.7-fold), Mn (7.9-fold) and Fe (7.4-fold). Seawater sampled from treatment III tanks exhibited 7.8-, 5.3- and 5.1-fold higher mean concentrations in Co, Fe and Mn, respectively, than treatment I (control). Although trace metal concentrations were on average approximately 1.5 times higher in treatment II than in treatment III tanks, significant differences were only observed in Ni (ANOVA, *p* < 0.0001), Co (ANOVA, *p* < 0.0001), Mn (ANOVA, *p* < 0.0001) and Cu (KW, *p* < 0.034).

**Table 1.** Seawater trace metal concentration in tanks maintained under three treatments (I – control, II – direct tephra exposure and III – indirect tephra exposure).

	treatment I – control	treatment II – direct exposure	treatment III – indirect exposure
element	*[nmol l^−^^1^]*	*[nmol l^−^^1^]*	*[nmol l^−^^1^]*
V	17.79 (± 2.57)	25.78 (± 3.51)	25.96 (± 3.96)
Mn	2.72 (± 0.37)	21.56 (± 2.96)	13.75 (± 0.74)
Fe	8.96 (± 2.22)	66.55 (± 16.20)	47.78 (± 38.30)
Co	0.16 (± 0.01)	1.57 (± 0.13)	1.26 (± 0.09)
Ni	2.75 (± 0.21)	29.15 (± 5.31)	10.00 (± 4.31)
Cu	2.87 (± 0.17)	19.68 (± 5.91)	9.39 (± 2.13)
Zn	104.26 (± 62.61)	189.72 (± 99.24)	107.04 (± 37.21)
Cd	0.75 (± 0.13)	1.61 (± 0.39)	1.51 (± 0.39)
Pb	0.19 (± 0.04)	0.66 (± 0.14)	0.59 (± 0.10)

*Note:* Each tank was sampled three times (on Mondays in the first, second and sixth weeks) at the end of the day. This gives *n* = 6 per treatment. Data are presented as mean (± s.d.).

### 3.3. Effective quantum yield

The monitored Φ_PSII_ increased constantly in all three coral species under treatment II (figure 2a), with yields plateauing at around 0.5–0.6 after approximately four weeks of tephra exposure. After six weeks, Φ_PSII end_ revealed significant differences between treatment I (0.18 ± 0.05), treatment II (0.51 ± 0.04) and treatment III (0.35 ± 0.04) nubbins of *S. pistillata* (ANOVA, *p* < 0.0001 in all cases; supplementary material, table S7). Similar observations were made for *T. reniformis* nubbins across all conditions with the highest Φ_PSII end_ for treatment II (0.57 ± 0.04) and treatment III (0.48 ± 0.05) nubbins, with significantly lower Φ_PSII end_ for control nubbins (0.29 ± 0.05). *P. damicornis* nubbins exposed to particulate tephra showed significantly higher Φ_PSII end_ (0.56 ± 0.03) compared to the control nubbins (0.24 ± 0.03; KW, *p* < 0.02), while no difference was observed between treatment I and treatment III and between treatment II and treatment III nubbins (supplementary material, table S7). All three species exhibited the lowest Φ_PSII max_ and Φ_PSII end_ (figure 2a) in treatment I.

**Figure 2.**
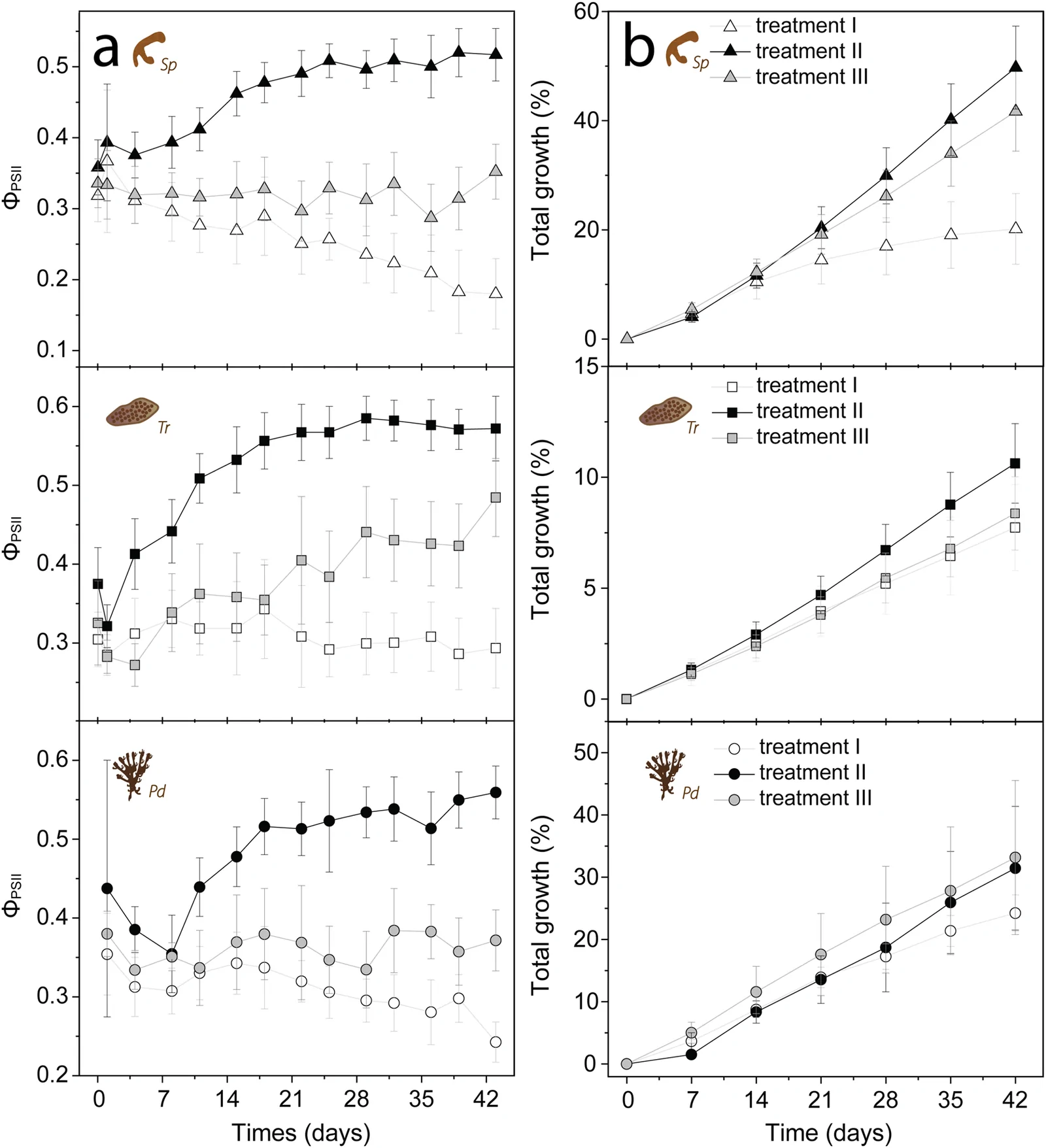
Time-resolved changes in monitored (a) Φ_PSII_ and (b) skeletal growth in three scleractinian species (*Stylophora pistillata (Sp)*, *Turbinaria reniformis (Tr)* and *Pocillopora damicornis (Pd)*) reared under three treatments (I – control (white), II – direct tephra exposure (black) and III – indirect tephra exposure (grey)); Φ_PSII_ was determined on *S. pistillata* nubbins (*n* = 8), *T. reniformis* (*n* = 6) and *P. damicornis* nubbins (*n* = 3) per treatment. Skeletal growth was measured as the percentage increase in weight relative to the initial weight for all nubbins (*S. pistillata* (*n* = 16), *T. reniformis* (*n* = 12) and *P. damicornis* (*n* = 3) per treatment). Data are presented as mean ± s.d.

### 3.4. Skeletal growth rate

Tephra exposure (either directly or indirectly) led to a significant increase in the growth rates of *S. pistillata* (figure 2b). Nubbins exposed directly to tephra (treatment II) gained on average 49.7 ± 7.6% in mass over six weeks, with a daily skeletal increase of 1.01 ± 0.13% d^−1^. Indirect tephra exposure (treatment III) led to increases in total nubbin growth of 41.7 ± 7.3% and daily skeletal growth of 0.87 ± 0.13% d^−1^. In all cases, these growth rates exceeded those of the control not exposed to tephra (treatment I; 20.2 ± 6.5% and 0.46 ± 0.13% d^−1^), with high statistical significance (ANOVA, *p* < 0.0001; supplementary material, table S9a). Total growth and daily growth rates differed significantly between treatment II and treatment III nubbins, with treatment II nubbins experiencing 1.2-fold enhanced growth (ANOVA, *p* < 0.01 and *p* < 0.016, respectively; figure 2b).

*T. reniformis* nubbins also experienced enhanced growth when exposed directly to tephra (10.6 ± 1.8% total growth and 0.25 ± 0.04% d^−1^; figure 2b), which was 1.3 times higher than the growth rate of treatment I nubbins (7.3 ± 1.9% total growth and 0.19 ± 0.04% d^−1^; ANOVA, *p* < 0.006 in both cases; supplementary material, table S9b). The skeletal growth parameters of nubbins indirectly exposed to tephra did not differ from treatment I nubbins (i.e. 8.4 ± 1.7%; ANOVA, *p* ≈ 0.50; supplementary material, table S9b), but compared to treatment II nubbins, their growth was significantly (0.8 times) lower (ANOVA, *p* < 0.019 in both cases). *P. damicornis* nubbin growth rates between individual treatments did not significantly differ (KW, *p* = 0.56; supplementary material, table S9c). They grew on average 24.2 ± 2.9% in treatment I, 31.4 ± 9.9% in treatment II and 33.2 ± 12.4% in treatment III during six weeks (figure 2b), which correspond to a daily growth rate of 0.54 ± 0.06% d^-1^ for treatment I, 0.68 ± 0.20% d^-1^ for treatment II and 0.71 ± 0.23% d^−1^ for treatment III.

### 3.5. Photosynthetic parameters

The weekly supply of particulate tephra (treatment II) enhanced overall the photosynthetic rates of *S. pistillata* and *T. reniformis* (figure 3 and supplementary material, fig. S4). Treatment II nubbins of *S. pistillata* exhibited enhanced net and gross oxygen production normalized per surface area and symbiont cell. Rates of *P*_net_ (supplementary material, fig. S4a,b) and *P*_gross_ (figure 3e,f) were significantly (*P*_net_: 6 to 8 times and *P*_gross_: 2 times) higher than measured in treatment I nubbins (all *p* < 0.002; supplementary material, table S9a). Treatment III nubbins had significantly higher *P*_net_ per surface area (0.23 ± 0.10 µmol h^−1^ cm^−2^; KW, *p* < 0.02) compared to control nubbins, while other photosynthetic rates were non-significant (*p* > 0.05). Similarly, nubbin respiration rates per surface area (approx. −0.32 ± 0.08 µmol h^−1^ cm^−2^) and symbiont cell (approx. −0.27 ± 0.09 pmol h^−1^ symbiont cell^−1^) were similar across all treatments (fig. S4c,d). Treatment II and treatment III nubbins differ in *P*_net_ and *P*_gross_ per symbiont cell, with treatment II nubbins experiencing 2.1-times higher *P*_net_ (ANOVA, *p* < 0.012; supplementary material, fig. S4) and 1.7-times higher *P*_gross_ (ANOVA, *p* < 0.005).

**Figure 3.**
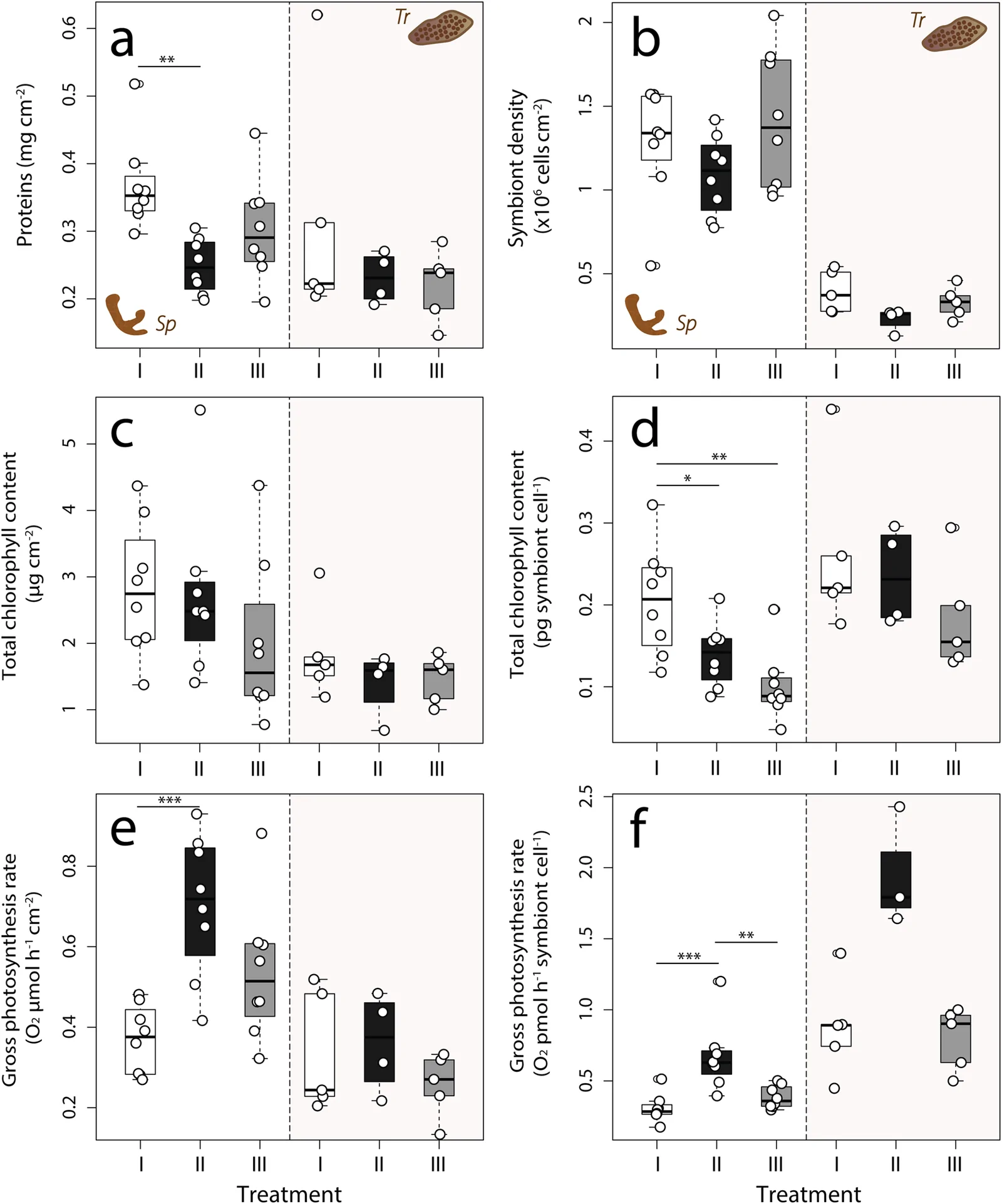
Physiological responses of *Stylophora pistillata (Sp)* and *Turbinaria reniformis (Tr)* reared under three treatments (I – control (white), II – direct tephra exposure (black) and III – indirect tephra exposure (grey)). Selected physiological parameters of *S. pistillata* (*n* = 8 per treatment, left) and *T. reniformis* (*n* = 6 per treatment, right) are expressed as (a) holobiont protein content, (b) symbiont density, total chlorophyll *a* and *c_2_* content normalized per (c) surface area and (d) symbiont cell and *P*_gross_ normalized per (e) skeletal surface area and (f) symbiont cell. Data are presented as box plots, where the black line represents the median, the box delimits the first and third quartiles, and the whiskers give the range within 1.5 of the interquartile range (IQR, defined as Q3–Q1). White circles with black borders represent individual data points. Statistically significant differences between treatments are indicated by asterisks (*), with the number of asterisks corresponding to level of significance (i.e. *p*-value > 0.05 (not significant), *p*-value ≤ 0.05 (*), *p*-value ≤ 0.01 (**) and *p*-value ≤ 0.001 (***)).

*T. reniformis* nubbins across the three treatments only differed significantly in *P*_net_ per symbiont cell (supplementary material, table S9b). The net photosynthesis rate of treatment II nubbins (0.89 pmol ± 0.33 h^−1^ symbiont cell^−1^) was significantly higher than in treatment I (0.34 pmol ± 0.15 h^−1^ symbiont cell^−1^; ANOVA, *p* < 0.0001) and treatment III nubbins (0.43 pmol ± 0.03 h^−1^ symbiont cell^−1^; ANOVA, *p* < 0.0001); *P*_gross_ and R rates were indifferent between treatments (supplementary material, table S9b).

RLC-derived *F*_v_/*F*_m_ and NPQ_max_ varied significantly between all three species under the three treatments (supplementary material, fig. S5 and table S7); *F*_v_/*F*_m_ values in treatment II corals (≈0.57 ± 0.02) were significantly higher than those in the controls (ANOVA, *p* < 0.0001 in all cases; supplementary material, table S7). Nubbins reared under treatment II also exhibited the lowest NPQ_max_, with 0.88 ± 0.09 for *S. pistillata*, 0.70 ± 0.10 for *T. reniformis* and 0.58 ± 0.10 for *P. damicornis* (supplementary material, fig. S5). The rETR_max_ was indifferent for *S. pistillata* and *T. reniformis* reared under the three treatments (*p* > 0.05). Only *P. damicornis* nubbins showed significant differences in treatment II (276.45 ± 7.71; ANOVA, *p* < 0.05) and treatment III (292.48 ± 36.21; ANOVA, *p* < 0.03) nubbins compared to treatment I nubbins (192.16 ± 27.86; supplementary material, fig. S5).

### 3.6. Symbiont density, protein content and chlorophyll content

Physiological parameters of *S. pistillata* nubbins reared under the three treatments showed distinct differences in protein content and chlorophyll *a* + *c_2_* content normalized per symbiont cells between individual treatments (figure 3a and supplementary material, fig. S6b), while *T. reniformis* nubbins did not exhibit statistically significant differences (supplementary material, table S9b). While the protein content in treatment II nubbins of *S. pistillata* was significantly lower than in the control (0.25 ± 0.04 mg cm^−2^ versus 0.37 ± 0.06 mg cm^−2^; ANOVA, *p* < 0.004), it did not differ between treatment II and treatment III nubbins and between treatment I and treatment III values (figure 3a). Deviations from control values were observed in the chlorophyll contents normalized per symbiont cell (figure 3d, supplementary material, fig. S6b,d), with treatment II and treatment III nubbins exhibiting significantly (0.68-times) lower chlorophyll *a* (approx. 0.9 ± 0.03 pg symbiont cell^−1^; ANOVA, *p* < 0.05) and total chlorophyll concentrations (approx. 0.12 ± 0.04 pg symbiont cell^−1^; ANOVA, *p* < 0.002) than in treatment I nubbins.

## 4. Discussion

### 4.1. Consistent photophysiological responses to direct tephra exposure across species

The trace metal enrichment in seawater with direct exposure to particulate tephra increased the photophysiological activity of all three hermatypic species. The benefits in *S. pistillata* and *T. reniformis* corresponded to significant increases in chlorophyll-fluorescence parameters (*F*_v_/*F*_m_, Φ_PSII max_ and Φ_PSII end_), *P*_net_ per surface area for *S. pistillata*, *P*_net_ per symbiont cell for *T. reniformis* and significant decreases in NPQ_max_ compared to non-exposed corals. Here, *P. damicornis* showed a uniform increase in rETR_max_ to both tephra supply forms, but only significant differences in *F*_v_/*F*_m_, NPQ_max_ and Φ_PSII end_ when directly exposed to tephra versus the control (supplementary material, table S7). Statistical indifference in results for *P. damicornis* nubbins compared to the other species could occur through the smaller sample size for *P. damicornis* (*n* = 3 per treatment) [52] compared to the other two coral species. This limitation in population size was due to the restricted availability of additional nubbins.

Upon direct exposure to particulate tephra, significant changes in the photosynthetic apparatus of Symbiodiniaceae were observed in Φ_PSII_ after a week for both *S. pistillata* and *T. reniformis* and after two weeks for *P. damicornis* compared to control nubbins (figure 2a). In coral ecophysiology, the Φ_PSII_ value, measured on the light-adapted nubbins, reflects the photochemical efficiency of PSII with temporal decreases in Φ_PSII_ linked to physiological stress [53]. In our experiment, however, all three species directly exposed to volcanic tephra showed logarithmically shaped increases in Φ_PSII_ (figure 2a) suggesting lower physiological stress. This is supported by *F*_v_/*F*_m_ levels in the three coral species significantly exceeding control values (supplementary material, fig. S5 and table S7). High *F*_v_/*F*_m_ levels indicate lower physiological stress given that stress is associated with low *F*_v_/*F*_m_ [54,55] and corresponding low ETR_max_ and high NPQ levels, with chronic photoinhibition of PSII linked to reduced coral reproduction and photosynthate translocation from symbiont to coral host [56,57]. The pattern of significantly higher *F*_v_/*F*_m_ and significantly lower NPQ_max_ levels suggests a more relaxed photophysiological state, in which adsorbed light is used more efficiently to drive photochemistry, with less need for energy dissipation via NPQ. Identical patterns were observed for chlorophyll-fluorescence-derived stress indicators in nubbins of *S. pistillata* exposed to lower concentrations of pristine volcanic ash (7.5 g week^−1^ tank^−1^) over six weeks [18].

The leaching of micronutrients from geogenic particles (i.e. desert dust [58,59] or volcanic ash [18,60]) has been directly linked to increases in the photochemical efficiency of Symbiodiniaceae. Among these micronutrients, Mn and Fe are quantitively the most important trace elements involved in oxygenic photosynthesis [61]. Direct additions of Mn to *S. pistillata* have been shown to enhance photosynthetic efficiency [62,63] and to help the coral host mitigate cellular stress during thermal stress [64]. Volcanic ash that released the highest concentrations of Mn and Fe had the strongest positive effect on phytoplankton growth rates [65], highlighting a potential link between seawater trace metal concentration and physiological enhancements as proposed by Förster *et al*. [27]. The improved performance observed in nubbins directly exposed to tephra, compared to indirectly exposed nubbins, may therefore be attributed to the elevated concentrations of trace metals in the seawater (table 1). This effect could be further amplified by particle ingestion and presumably enhanced leaching owing to enzymatic and pH differences between seawater and the coelenteric fluid within the coral tissue [66]. Altogether, these findings suggest that the Symbiodiniaceae are limited in micronutrients for an optimal photosynthetic functioning, and that weathered tephra, similar to pristine ash [18], leach hormetic (beneficial, low-level) concentrations of essential trace metals. Metal toxicity, induced by harmful concentrations of metals [55], can enhance radical oxygen species production, especially in Symbiodiniaceae [67,68], harming the coral holobiont when anti-oxidant capacities are overwhelmed [69]. However, even for more reactive volcanic ash collected immediately after the eruption of the La Soufrière volcano, no difference was observed in lipid peroxidation between controlled and ash-exposed *S. pistillata* nubbins [18]. In addition, metal toxicity would affect the organism's photochemistry (i.e. decrease in *F*_v_/*F*_m_ [55]), which we did not observe (supplementary material, fig. S5). Hence, the increase in Φ_PSII_ suggests physiologically better performance in nubbins exposed to weathered tephra.

The consistent physiological responses observed for tephra exposure are significant, given that the three coral species exhibit distinct microbial/Symbiodiniaceae assemblages with species-specific metal requirements [70,71]. Furthermore, significant increases in the skeletal growth for *S. pistillata* and *T. reniformis* nubbins hint at optimized translocation of energy-dense photosynthates from symbionts to coral host [57]. Assimilation and translocation rates of inorganic carbon are influenced by Symbiodiniaceae genotypes (traditionally ‘clades’) [57,72]. Differences in Symbiodiniaceae clades are also linked to different photophysiological performance [57,73]. Although *S. pistillata*, *T. reniformis* and *P. damicornis* host different genotypes of Symbiodiniaceae, with *Symbiodinium* Clade A more common in lab-grown *S. pistillata* [74] and *Cladocopium* Clade C predominantly associated with *T. reniformis* [74] and cultured *P. damicornis* [75], their photobiological response to tephra-supplied metals appears similar.

### 4.2. Tephra-supplied micronutrient budgets and the effects of exposure types

All measured trace metals were significantly enriched in the tephra-exposed seawater samples compared to the non-exposed treatments except for Zn (table 1 and supplementary material, table S4), which highlights a substantial input of trace metals from weathered tephra (table 1 and supplementary material, table S4). While bio-relevant elements (micronutrients), such as Mn and Fe, were released in large quantities (increasing seawater concentrations by approx. 5–8-fold), potentially toxic elements, such as Cd and Pb, were also markedly higher in direct and indirect tephra-exposed seawater compared to non-exposed seawater. However, given the analytical challenges associated with Cd and Pb (§2.3), those results need to be treated with caution.

The total dissolved metal concentrations differed between exposure types, with on average approximately 1.5-times higher concentrations in directly exposed versus indirectly exposed samples. Significant differences between exposure types were observed for Ni, Mn, Co and Cu, whereas Pb, Cd and Fe were released to a similar extent between exposure types (*p* > 0.05; supplementary material, table S4).

Note that corals directly exposed to tephra probably experienced higher overall metal concentrations throughout the week than indirectly exposed corals. This is because in treatment II the corals were continuously exposed to tephra from addition on Monday morning until cleaning on Friday evening. Comparatively, in treatment III, the peristaltic pump connecting the leachate reservoir with the exposure tanks operated 9 h per day, meaning that exposure was not continuous between Monday morning and Friday evening.

When comparing the leaching potential between different tephra types from St. Vincent (i.e. weathered versus pristine tephra; table 2) under an assumption of a non-limited leaching process, notable and non-uniform differences in the released metal contents occur. Pristine volcanic ash generally leaches higher amounts of Mn and Zn, whereas the weathered tephra releases higher amounts of Fe, Ni, Cd, Pb and V into seawater (table 2). The elevated Pb and V content in seawater exposed to weathered tephra compared to the pristine volcanic ash is probably due to scavenging by reactive ash surfaces [17]. In both cases, leachate concentrations from pristine and weathered tephra remain largely below the global median values, yet are within the global range presented in Ayris & Delmelle [76] (table 2). Weathering differences among tephra types can influence trace metal release and, in turn, affect physiological responses, although the overall organismal performance remained largely comparable in tephra-exposed nubbins of *S. pistillata*.

**Table 2.** Comparison of leached trace metal concentrations [mg kg^−1^ tephra] from different La Soufrière tephra collected on St. Vincent and global median concentrations.

	treatment II direct tephra exposure	treatment III indirect tephra exposure	pristine volcanic ash	global median concentrations
element	*[mg kg^−^^1^ tephra]*	*[mg kg^−^^1^ tephra]*	*[mg kg^−^^1^ tephra]*	*[mg kg^−^^1^ tephra]*
V	0.244	0.250	−0.01	0.089
Mn	0.621	0.364	4.808	20
Fe	1.930	1.301	0.125	21
Co	0.050	0.039	0.031	0.186
Ni	0.929	0.255	0.076	0.5
Cu	0.641	0.249	0.878	5
Zn	3.352	0.109	8.911	3.6
Cd	0.058	0.051	0.007	0.053
Pb	0.059	0.049	−0.001	0.114
**ratio (g : ml)**	1:600*	1:600*	1:100	various
**leaching solution**	seawater	seawater	seawater	water
**duration**	9 h	9 h	24 h	1 h
**experiment**	coral culture	coral culture	leaching	leaching
**reference**	this study	this study	Förster *et al*. [17]	Ayris & Delmelle [76]

*Note*: Measured seawater metal concentrations (table 1) were converted into mass of elements leached per mass of supplied tephra as outlined in Förster *et al*. [17].

* Corresponding to 50 g tephra: 30 l seawater.

### 4.3. Tephra run-off as important contributor to the island mass effect

Trace metals, which can act as essential micronutrients for marine primary producers, are important but less appreciated contributors to the island mass effect [77,78], which describes an increased primary productivity in proximity to islands, with phytoplankton biomasses increased by up to 86% compared to oligotrophic oceanic conditions [79]. In corals, it has been shown that, for example, *Acropora muricata* colonies in shoreline reefs experienced higher photochemical efficiency (NPQ_max_ and rETR_max_) compared to open reef colonies [80]. Multiple factors can account for this, including biotic inputs (e.g. faeces-derived nutrients [81,82]) and geological factors such as submarine groundwater discharge, slope angle and terrestrial sources [79], all of which increase the nutrient availability near islands. While volcanogenic metal discharge from subaerial volcanism has been hypothesized to be a major contributor for element cycling in ocean biogeochemistry [83], our findings indicate that remobilized and reworked tephra can act as natural metal supplementation for coral reefs in proximity to volcanic islands.

The consistent physiological response following exposure to volcanic tephra, which was collected *in situ* from the volcano and added without further sample preparation into the tanks, can help us better understand the importance of sediment input from tropical volcanic islands in shaping coastal water chemistry. In laboratory settings, exposure to pristine [18] and weathered tephra (this study), produced by the explosive eruption of the La Soufrière volcano, stimulated primary productivity by leaching bioavailable metals in hormetic concentrations, which, to a lesser degree, has also been observed in desert dust exposure experiments [58,59]. While the tephra leaching potential is dependent on, for example, grain size, surface area, bulk and mineralogical composition of the material [84,85], we observed similar physiological effects at 250 mg volcanic ash l^−1^ week^−1^ [18] and 50 g tephra per tank (1.67 g tephra l^−1^ week^−1^) in nubbins of *S. pistillata.* Although weathered and unsieved tephra has a different metal input than freshly erupted ash (per unit mass), influenced by different surface area per unit mass and amount of reactive components [23], the supplied trace metal concentrations at approximately 0.57 kg m^−2^ tephra loading resulted in a similar photobiological response across the three different scleractinian species in this study.

Given the micronutrient-driven physiological responses described above, we suggest that tephra-enriched terrestrial and river run-off can alter coastal biogeochemistry in ways that support coral–algae symbiosis and overall benefit organismal health, primarily by supplying Mn and Fe, which are linked with improved coral vitality and stress resilience [59,62,63,86]. Nevertheless, the contribution of tephra-derived micronutrients to coastal ecosystems can have multiple effects on reef scale, thus causing/promoting an imbalance in the dominance of reef communities [87,88]. Micronutrient enrichments can promote increasing phytoplankton abundance [88], thus increasing pressures of interspecific benthic competition [87]. It must be tested via mesocosms and field studies how tephra input around volcanic islands affects coastal water geochemistry and how it translates to changes in coral physiology and coral reef assembly.

## 5. Conclusion

In this coral culture study, tephra-derived trace metal input enhanced the photophysiological activity of all three scleractinian corals. Long-term and probably altered tephra, functions as a ‘metal cocktail’ similarly to volcanic ash [18] and desert dust [58,59] by supplying a range of essential trace elements to oligotrophic reef waters. This input supports coral metabolism by enhancing primary productivity of Symbiodiniaceae and, thus, strengthens the symbiotic relationship between algae and coral host. Although most physiological parameters (i.e. symbiont densities, total chlorophyll content per surface area and respiration rates) were indistinguishable between *S. pistillata* and *T. reniformis* nubbins reared for six weeks under the three experimental treatments (control, direct and indirect tephra exposure), significant increases were observed in skeletal growth rates, *P*_net_ and chlorophyll-fluorescence parameters (i.e. *F*_v_/*F*_m_, Φ_PSII_ and NPQ_max_) under tephra exposure. Particulate and washed out tephra run-off is probably an important contributor to the near-shore trace metal concentrations in the coastal reef environment, which, according to the results of this study, may support coral photobiology and, thus, enhance coral resilience. This study focused specifically on tephra from St. Vincent as an initial step towards understanding these mechanisms, but it would be valuable to repeat similar experiments with a range of physicochemical tephra characteristics to better assess the generality of these responses across volcanic systems.

## Data Availability

Supplementary material and the original data that support the findings of this study are available in Mendeley Data with the identifier [49].
